# Activity Counts: The Effect of Swimming Activity on Quantity Discrimination in Fish

**DOI:** 10.3389/fpsyg.2012.00484

**Published:** 2012-11-12

**Authors:** Luis M. Gómez-Laplaza, Robert Gerlai

**Affiliations:** ^1^Department of Psychology, University of OviedoOviedo, Spain; ^2^Department of Psychology, University of Toronto MississaugaMississauga, ON, Canada

**Keywords:** quantity discrimination, continuous variables, swimming activity, angelfish, shoal choice, numerical cognition

## Abstract

Human infants and non-human animals can discriminate the larger of two sets of discrete items. This quantity discrimination may be based upon the number of items, or upon non-numerical variables of the sets that co-vary with number. We have demonstrated that angelfish select the larger of two shoals of conspecifics without using inter-fish distance or space occupied by the stimuli as cues. However, density appeared to influence the choice between large shoals. Here, we examine the role of another non-numerical cue, swimming activity of the stimulus fish, in quantity discrimination by angelfish. To control this variable, we varied the water temperature of the stimulus aquaria or restricted the space occupied by each fish in the stimulus shoals. We used the previously successfully discriminated contrasts consisting of large (10 vs. 5) and small (3 vs. 2) shoals. We also studied whether more active or less active shoals are preferred in case of equally sized shoals (10 vs. 10, 5 vs. 5, and 3 vs. 3). When differences in stimulus fish activity were minimized by temperature manipulation we found angelfish to prefer the larger shoal in the 3 vs. 2 comparison, but not in the 10 vs. 5 comparison. When activity was controlled by space restriction, angelfish preferred the larger shoal in both numerical contrasts. These results imply that the overall activity level of the contrasted shoals is not a necessary condition for small shoals discrimination in angelfish. On the other hand, the results obtained for the large shoals, together with results obtained in the control treatments (equal numerical contrasts and differing activity levels), suggest that activity is a sufficient condition for discrimination when large shoals are involved. Further experiments are needed to evaluate the influence of other continuous variables, and to assess whether the mechanisms underlying performance are comparable to those suggested for other animals.

## Introduction

In the past decades a wealth of studies have provided evidence suggesting that human infants and other animal species possess non-verbal numerical competence encompassing a diversity of categories (Gallistel and Gelman, 2000; Feigenson et al., 2004; Hauser and Spelke, 2004). The study of numerical competences is of importance in comparative research because of the potential implications for understanding the evolutionary origins and development of such capabilities. For example, a growing number of studies indicate that quantity discrimination, the ability to distinguish between sets of discrete elements of different numerical size is a robust phenomenon across a large number of animal species. This capability has been shown not just in human and non-human primates (e.g., Hauser et al., 2000; Xu, 2003; Cantlon and Brannon, 2006; Hanus and Call, 2007; Evans et al., 2009) where most work has been conducted, but also in other mammalian species such as elephants (Irie-Sugimoto et al., 2009), bears (Vonk and Beran, 2012), dolphins (e.g., Kilian et al., 2003), horses (Uller and Lewis, 2009), coyotes (Baker et al., 2011), voles (Ferkin et al., 2005), dogs (West and Young, 2002; Ward and Smuts, 2007), cats (Pisa and Agrillo, 2009), and rats (Capaldi and Miller, 1988), birds (e.g., Emmerton and Renner, 2006; Rugani et al., 2008; Al Aïn et al., 2009; Bogale et al., 2011; Fontanari et al., 2011), fish species (e.g., Buckingham et al., 2007; Bisazza et al., 2010; Agrillo et al., 2011; Piffer et al., 2012), amphibians (Uller et al., 2003; Krusche et al., 2010), and even in invertebrates (e.g., Gross et al., 2009; Reznikova and Ryabko, 2011). Findings in this large variety of organisms provide good evidence to support the idea that the ability to discriminate between differently sized quantities has ancient evolutionary roots. This may not be surprising considering that the ability to quantify may have an adaptive role with potential advantages in functionally different contexts. These include evaluation of food sources (e.g., Creswell and Quinn, 2004; Bar-Shai et al., 2011), parental investment (e.g., Lyon, 2003), threats, and social interactions (e.g., Benson-Amram et al., 2011; Bonanni et al., 2011), as well as protection from predators and from sexually pursuing males (e.g., Hager and Helfman, 1991; Agrillo et al., 2007).

In several studies, however, numerical information was confounded with a variety of other variables which co-vary with item number. Although in nature individuals may attend simultaneously to both number and continuous quantities (Davis and Perusse, 1988), the failure to control for continuous non-numerical properties of the stimuli such as perimeter, density, surface area, visual extent, or movement makes it difficult to evaluate whether numerical competence indeed exists in all species studied. Thus, whether individuals discriminate between discrete quantities of items relying solely on number or they respond to a variety of continuous variables is still a matter of debate (Mix et al., 2002).

This question has mostly been investigated in human infants and non-human primates, where experiments have specifically been designed to disentangle the influence of these confounds by using stringent controls for non-numerical continuous variables. Apparently contradictory results have been obtained. Some studies report that infants and non-human primates respond to continuous variables instead of number, mainly when discriminating between small numbers of elements (Clearfield and Mix, 1999; Feigenson et al., 2002; Stevens et al., 2007). Other studies, after controlling for continuous extent, have found that individuals base their discrimination on numerical differences (Feigenson and Carey, 2003; Xu, 2003; Xu et al., 2005; Beran, 2007; Cantlon and Brannon, 2007; Tomonaga, 2008). The picture emerging from these studies suggests that both infants and non-human primates can rely spontaneously on number even when continuous variables are available, indicating that the use of number for discrimination is not a last resort strategy for them (Cantlon and Brannon, 2007; Cordes and Brannon, 2009). Likewise, it appears that infants respond to number rather than continuous extent when presented with object sets of contrasting properties (color, pattern, texture) and rely on continuous extent over number when identical objects are presented (Feigenson, 2005). In line with this, Beran et al. (2008) conclude that chimpanzees preferentially attended to number over continuous variables or vice versa depending on the task and/or experimental conditions. Nevertheless, research has not provided a clear account of under what condition animals may rely on either number or continuous variables. Although in most studies controlling for numerous factors has been attempted, it is not possible to completely disregard the possibility that the discrepancies arose as a result of the effect of the subjects’ experience or other ontogenetic factors (but see Feigenson et al., 2004).

In non-primate animals, research on whether individuals discriminate between two sets of stimuli on the basis of number or continuous dimensions is rather scarce. Nevertheless, empirical studies with birds, mainly pigeons (e.g., Xia et al., 2001; Machado and Keen, 2002; Emmerton and Renner, 2006; Scarf et al., 2011) and chicks (e.g., Rugani et al., 2008, 2009, 2010), resulted in findings comparable to those obtained with human infants and primates. Similarities have been found in newborn chicks even in the use of continuous extent or number: newborn chicks will also discriminate between set of objects based on continuous extent over number if objects are homogeneous rather than heterogeneous (Rugani et al., 2010). In other animals this issue has not generally been systematically investigated and findings indicate that whereas some species when confronted with quantity discrimination tasks use the type of information provided by continuous variables (e.g., Pisa and Agrillo, 2009; Krusche et al., 2010) other species have been shown to use number as the relevant cue (e.g., West and Young, 2002; Kilian et al., 2003; Gross et al., 2009; Bogale et al., 2011).

In fish, this issue has also received only little attention. Although fish can discriminate between groups (shoals) of conspecifics of different numerical size (Krause et al., 1998; Bradner and McRobert, 2001; Binoy and Thomas, 2004; Agrillo and Dadda, 2007; Agrillo et al., 2007, 2008a; Buckingham et al., 2007; Frommen et al., 2009; Piffer et al., 2012), generally no control for continuous variables has been attempted. To date the only comprehensive approach to unravel the cues that guide fish in selection of numerically different shoals has been carried out in mosquitofish, *Gambusia holbrooki*, (Agrillo et al., 2007, 2008b, 2009, 2010, 2011) and evidence indicates that this fish species is able to discriminate the larger of two shoals solely on the basis of number (Dadda et al., 2009), although perhaps not all factors were controlled. For example, in this study the requirement for the experimental fish to see only one stimulus fish at a time may not have been met due to the large visual field of the study species that likely allowed these fish to view more than a single stimulus fish at a time in the employed experimental set up. Studies in angelfish (*Pterophyllum scalare*) also indicate that this cichlid species can discriminate between shoals of conspecifics of different size both when large shoals (≥4 fish) and when small shoals (<4 fish) are contrasted (Gómez-Laplaza and Gerlai, 2011a,b). In these studies angelfish always showed preference for the larger shoal when placed in a potentially threatening novel environment, presumably because larger shoals provide greater safety. But the question whether this discrimination was based upon numerical abilities of the angelfish or perception of a co-varying quantitative variable remained unaddressed. In a subsequent study (Gómez-Laplaza and Gerlai, 2013 in press) we began a systematic analysis of the potential non-numerical factors affecting shoal choice decisions in angelfish. Our results show that density of the shoals did affect the selection when angelfish were comparing large shoals (10 vs. 5 fish), but not when they were choosing between small shoals (3 vs. 2 fish). Inter-fish distance and space occupied by the stimulus shoals were found to have no significant effect in test fish’s preference when both large (10 vs. 5 fish) and small shoals (3 vs. 2 fish) were contrasted.

In the present study, to gain a better understanding of the potentially intervening variables that affect decision making we decided to assess the potential role played by another non-numerical cue. This allows further investigation of whether angelfish posses a strict form of numerical competence or use other quantitative cues to guide their responses. Specifically we analyze the influence of swimming activity of the stimulus shoals on the ability of angelfish to discriminate between two shoals of different numerical size simultaneously presented. The amount of movement in the larger shoal is likely to be greater than in the smaller shoal. More active shoals may provide a more salient stimulus for a solitary fish seeking a shoal with which to associate. Consequently, angelfish could respond to how much movement is present within each of the shoals. In fact, swimming activity has been shown to influence shoal association decisions in fish (e.g., Pritchard et al., 2001; Gómez-Laplaza, 2006; Agrillo et al., 2008b; Harcourt et al., 2009), and movement of the stimuli was shown to affect quantity discrimination in other animal species too (e.g., Krusche et al., 2010).

Here two numerical comparisons were used: 10 vs. 5 fish (large numbers in both shoals) and 3 vs. 2 fish (small numbers in both shoals). These contrasts have previously been found to be reliably discriminated by angelfish which chose the larger of the two contrasted shoals (Gómez-Laplaza and Gerlai, 2011b, 2013 in press). In the present study, we controlled for swimming activity by minimizing the potential difference in total level of activity of the shoals to be compared. This was achieved either by lowering the temperature of the water of the aquarium in which the shoals with the larger number of members was presented while increasing the temperature of the shoals with the smaller number of members (Experiment 1), or by keeping the stimulus fish in small individual compartments, thus allowing little swimming (Experiment 2). We also performed the opposite manipulation and kept the number of contrasted shoal members constant while making the equally sized contrasted shoals differ in their activity levels.

## Materials and Methods

### Subjects and housing conditions

Wild type juvenile angelfish (*Pterophyllum scalare*, 2.8–3.0 cm standard length) were obtained from local commercial suppliers. Since differences in color morph of the subjects can influence results (Gómez-Laplaza, 2009) only fish from the same color morph were used. Likewise, only juveniles of this sexually monomorphic species were studied so as to eliminate possible confounding effects arising from courtship or agonistic/territorial interactions. The fish were housed in glass holding aquaria (length × width × depth: 60 cm × 30 cm × 40 cm) in groups of 18–20 and were allowed a minimum of 2 week acclimation period before behavioral testing.

Test fish and stimulus fish (which were used to elicit test fish behavior) were randomly chosen and were housed separately, with no visual and olfactory communication being possible between fish in the separate aquaria. Aquaria were filled with dechlorinated tap water kept at 25°C using thermostat-controlled heaters. Each aquarium was illuminated by a 15 W white fluorescent tube on a 12:12 h light:dark cycle, with lights on at 08:30 h. External filters continuously cleaned the aquaria, which were provided with a 2 cm gravel substratum. The fish were fed commercial fish food (JBL GALA, JBL GmbH & Co. KG, Neuhofen, Germany) twice daily, at 10.00 h and at 18.00 h.

### Experimental apparatus

The experimental apparatus to assess spontaneous shoaling preference in binary choice tests was similar to what we used in previous studies (Gómez-Laplaza and Gerlai, 2011a,b). It consisted of a test aquarium with one stimulus aquarium positioned at each end. The test aquarium was identical in all respects to the holding aquaria and was maintained under the same conditions, as also were the stimulus aquaria. The stimulus aquaria were of smaller dimensions (30 × 30 × 40 cm depth) but the side facing the test aquarium was of the same size as the short lateral sides of the latter (30 × 40 cm). The test aquarium and stimulus aquaria were illuminated with a 15 W white fluorescent light tube. A divider isolated a 10 cm compartment in the stimulus aquaria where the stimulus shoals were presented. In the other part of the stimulus aquaria, the stimulus shoals were placed before preference tests commenced. Except for the front, all exterior walls of the aquaria that were not adjacent to other aquarium walls were lined with white cardboard to prevent the fish from being influenced by external visual stimuli. Removable opaque white barriers placed outside the two end sides of the test aquarium were used to visually isolate the latter from the stimulus aquaria and these barriers were removed when preference tests commenced.

Five vertical lines drawn on the front and back walls of the test aquarium at a distance of 10 cm divided the test aquarium into six equal zones and facilitated measurements of the test fish’s movements and position. The two 10 cm zones closest to the stimulus aquaria were considered as the preference zones. At least three-quarters of the body length of the fish had to be within the boundary for the fish to be included in a particular zone. Swimming activity of test fish was measured as the frequency (number of times) with which fish crossed the lines drawn on the walls of the aquarium during the tests.

### General experimental protocol: Preference tests

The experimental procedure was also similar to what has been described previously (Gómez-Laplaza and Gerlai, 2011a,b). In each trial a single test angelfish was given a choice between two numerically different shoals of conspecifics presented simultaneously and positioned in the stimulus aquaria on opposite sides of the test aquarium. The chosen number of fish that served as stimulus shoals were taken at random from the stimulus fish holding aquaria and were gently placed into the part of the stimulus aquaria not occupied by the stimulus compartment. To control for any potential side bias the allocation of the shoals to the stimulus aquaria was initially determined at random and then counterbalanced across trials. All fish were gently handled using dip netting and transferred between aquaria in small Perspex containers to minimize possible handling stress. In addition, all fish were allowed a 15 min acclimation period in the new aquaria (see below). Trials took place 15–30 min after feeding in the morning (i.e., they started around 10:15–10:30 h) when the stimulus shoals where gently transferred into the part of the stimulus aquaria not occupied by the stimulus compartment. Test fish were randomly selected from a test fish holding tank, and were introduced singly to the center of the test aquarium. Fish were allowed to swim freely with the barriers between aquaria removed, so they could see the 10 cm compartments where the stimulus shoals would be presented. This acclimation period in the absence of stimulus shoals lasted for 15 min and also allowed stimulus shoals to settle in the respective stimulus aquaria. At the end of this period, the barriers between aquaria were replaced and the stimulus shoals were gently placed into the 10 cm compartment. Test fish were placed in the center of the test aquarium via a transparent, open-ended, plastic cylindrical start box (7 cm diameter), where they remained for 2 min. During this time, the opaque white barriers between the aquaria were removed to reveal the stimulus shoals, thus allowing the confined test fish to view the stimulus shoals at both sides of the test aquarium. The start box was then gently raised and the test fish released. Shoaling behavior, recorded over a 15 min period, was defined as the time spent by the test fish in the 10 cm preference zones, i.e., within 10 cm from the wall adjacent to the stimulus shoal aquaria on either side. Behavioral responses of the test fish were recorded with a video camera (Sony video Hi8, model CCD-TR750E) concealed behind a blind. The recordings were later replayed for analysis.

At the conclusion of the recording session, the barriers between aquaria were replaced and the positions of the stimulus shoals were interchanged between stimulus aquaria to control for any potential directional bias (except for Experiment 1 in which replacing the water of the stimulus aquaria at different temperatures was not practical). After a second 15 min settling interval, another 15 min observation period was run with the same test fish following the same procedure as described above. After the second observation period, the aquaria were emptied and cleaned before being replenished with dechlorinated tap water. In the experiments individual fish were tested only once, and none of the fish in the stimulus shoals were used as test fish. Within each experiment, the order of testing was randomized according to different treatment conditions. Stimulus shoals were rearranged after each session, so that each test fish was exposed to a different stimulus fish set. The fish were returned to the suppliers at the end of the study. The experiments described here comply with the current laws of the country (Spain) in which they were performed (ref.: 13-INV-2010).

### Experiment 1: Control for swimming activity in large (10 vs. 5) and small (3 vs. 2) shoals by manipulating water temperature

The aim of this experiment was to examine whether the preference previously shown by angelfish for the larger shoal, in both 10 vs. 5 and 3 vs. 2 contrasts, could have been influenced by the swimming activity of the stimulus fish within the shoals regardless of shoal numerical size. One common way of controlling for swimming activity is by varying water temperature. Because teleost fishes are ectothermic, swimming activity is generally linked to water temperature as body temperature influences metabolic efficiency for many physiological processes (e.g., Bennett, 1990). Therefore, it is possible to modify the swimming activity of angelfish by increasing or decreasing water temperature, a procedure that has been used in choice situations in a number of fish species (Pritchard et al., 2001; Agrillo et al., 2008b). Angelfish is a gregarious Amazonian cichlid species which is widely distributed over a vast area and is adapted to a highly variable natural environment (White, 1975). In the laboratory, angelfish has also been shown to be able to live in a broad range of temperatures (Pérez et al., 2003). Here, initially to test whether indeed swimming activity of angelfish can be manipulated through temperature, we used three thermostat-controlled water temperatures, 21, 25, and 29°C, that are within the temperature tolerance limit for this species. First, three groups of 14 fish were placed each in one holding aquarium whose temperature was 25°C. In one of the aquaria the temperature was gradually raised 1°C per day, for 4 days, until a temperature of 29°C was reached, whereas in other aquarium the temperature was gradually lowered 1°C per day, also for 4 days, until a temperature of 21°C was reached. Fish in the remaining aquarium were kept at 25°C. Once the final temperatures were reached, groups were maintained at these temperatures for 10 days. Then, fish of each of the groups were individually transferred to a new aquarium (60 cm × 30 cm × 40 cm) where swimming activity was measured. The water temperature of the new aquarium was adjusted to the corresponding temperature of the previous holding aquarium of the fish to be tested (21, 25, or 29°C). After a 15 min acclimation period, fish locomotor activity was recorded for 15 min with the video camera. We quantified swimming activity by counting the number of cells entered (5 × 6 cm high) of a grid drawn on the frontal wall of the new aquarium. Each fish was used only once.

Thereafter, to control for the potential effects of swimming activity on quantity discrimination, we gave test fish the choice between two shoals of different numerical size presented at two different water temperatures. These preference tests were carried out as indicated above. When testing preference between two large shoals (10 vs. 5), fish in the larger stimulus shoal were presented in the stimulus compartment with water at 21°C (the same temperature as their corresponding holding aquarium), whereas fish in the smaller shoal were presented in water at 29°C (the same temperature as their corresponding holding aquarium). Similarly when testing preference between shoals in the small number range (3 vs. 2): the larger shoal was presented in water at 21°C, whereas the smaller shoal at 29°C. The water temperature in the test aquarium, where the test fish were introduced, was at 25°C (the same temperature as test fish holding aquarium). The position of the stimulus shoals was counterbalanced across subjects. Fourteen fish were observed for each of the two sets of choices (i.e., a total of 28 experimental fish). To ensure that swimming activity was equated between the stimulus shoals, while the focal fish were being tested we also recorded, with an additional concealed video camera (Sony Handycam HDR-XR160E), the activity of the stimulus fish in their respective compartments. Recordings were carried out with the camera angled to allow activity to be observed, and were alternated between the two stimulus compartments, thus obtaining seven recordings of each stimulus shoal in each contrast. For two randomly selected fish in each shoal, we measured the number of cells crossed (5 × 6 cm high) by these stimulus fish over the 15 min period. The number was averaged for the two fish to give a mean value for each stimulus shoal size. Given the difficulty of monitoring fish in the 10 fish shoals, the fish to be observed were identified by previously making small cuts on some of their fins. This process took less than 30 s with fish recovering immediately and no effect on their later behavior was observed.

A set of control experiments were also performed. These consisted of exposing test angelfish to pairs of shoals composed of the same number of fish: 10 vs. 10, 5 vs. 5, and 3 vs. 3 but presented in different water temperature (i.e., one of the shoals was placed in water at 21°C, slow moving, whereas the other, of equal numerical size, was placed in water at 29°C, fast moving). Fourteen fish were observed in each set of choices, giving a total of 42 fish tested.

### Experiment 2: Control for swimming activity in large (10 vs. 5) and small (3 vs. 2) shoals by restricting swimming of the fish in the stimulus shoals

**Figure 1:**
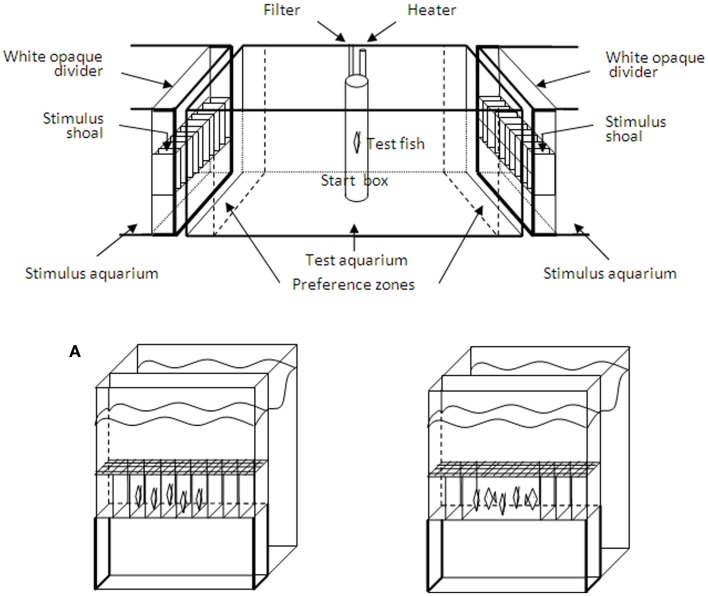
**The experimental apparatus with the central test aquarium and the two stimulus aquaria**. In the latter, removable opaque dividers were used to delimit a 10 cm compartment where the stimulus shoals were placed. Opaque barriers were used to visually isolate the two stimulus aquaria from the test aquarium. These barriers were removed when the preference test commenced. The time spent by the test fish within 10 cm of the stimulus shoals (preference zones) was recorded. The frames delimiting 10 small identical sectors where fish of the stimulus shoals were placed in Experiment 2 are also shown. Figure is not to scale. **(A)** The stimulus aquaria with the stimulus compartments utilized in Experiment 2. To control for the overall swimming activity of the shoals, the stimulus compartments were divided into 10 sectors by transparent partitions and each fish of the stimulus shoals was individually placed into the adjacent sectors. In the control treatments (one shoal more stationary and the other moving), the central partitions were removed and fish in one of the shoals were allowed to swim freely in that space (illustrated in the case of 5 vs. 5 fish in the Figure). Presentation of the stimulus shoals in the stimulus compartments was balanced between the two stimulus aquaria.

In this experiment we used another way of controlling for the potential effect of swimming activity on quantity discrimination in an attempt to further clarify the role of this variable on performance of angelfish. It consisted of equating activity in the shoals by ensuring that all stimulus fish had a similar level of activity. Two removable transparent Plexiglas frames delimiting 10 small identical sectors (length × width × depth: 3 cm × 10 cm × 13 cm) were constructed and introduced into each stimulus compartment (Figure 1). Stimulus fish were confined in these small sectors that allowed little movement, thus providing control over movement and orientation. The stimulus shoals were presented in midline of the aquaria. When testing preference between large shoals, each single stimulus fish of the 10 fish shoals was confined into each of the 10 separate small sectors, whereas each of the fish of the five fish shoals was confined into each of the five central small sectors of the frames. Similar procedure was followed when the test fish were presented with a binary choice between three fish shoals and two fish shoals, stimulus shoals being now confined in the central small sectors. Note that by positioning the stimulus fish in this way, density and inter-fish distance was also equated; therefore these three non-numerical continuous variables were simultaneously controlled. Fourteen test fish were observed for each of these two sets of choices (i.e., a total of 28 experimental fish were tested).

In addition, to control for general effects of swimming activity we ran a further set of control experiments. The treatments consisted of presenting pairs of equally sized stimulus shoals in which fish in one of them were confined into each of the small sectors, thus remaining stationary, whereas fish of the other stimulus shoals were allowed to swim within the entire space occupied by the fish in the confined shoal. This was done by removing the corresponding partitions delimiting the sectors in the frame (see Figure 1A, as an example). Thus, the overall space occupied by the contrasting shoals was the same because the outermost walls were kept in position, but in one of the shoals the individuals could move around in the entire space instead of being confined into the small sectors. Three control conditions were employed: 10 vs. 10 fish, 5 vs. 5 fish, and 3 vs. 3 fish, each including 14 experimentally naive test fish (i.e., a total of 42 fish were observed).

### Statistical analysis

The time spent in the preference zones was recorded as a measure of each test fish’s social preference for a particular stimulus. We calculated a preference index for each test fish as follows: time spent in the preference zone near the larger stimulus shoal was divided by the total time spent shoaling (i.e., the time spent within 10 cm from either stimulus shoals). A preference index equaling 1 would indicate complete preference for the larger shoal, whereas an index value of 0 would indicate complete preference for the smaller shoal. In the control treatments, with equal number of fish in the contrasting shoals, the preference index was calculated similarly but the numerator referred to the warm-water shoal (Experiment 1) or free-swimming shoal (Experiment 2). A one sample two-tailed *t*-test was used to compare the observed proportions against a chance value of 0.5 (null hypothesis). The proportions were normally distributed. Statistical probabilities reported are two-tailed. The null hypothesis was rejected when its probability (*P*) was less than 0.05.

The effect of water temperature on swimming activity was investigated with one-way ANOVA for independent samples. In case of a significant effect, Tukey Honestly Significant Difference (HSD) *post hoc* multiple comparison test was performed to determine where significant differences lay.

In Experiment 1, occasionally test fish did not enter both preference zones during the test. When this occurred the subjects were excluded and replaced by another fish. Five subjects (7%) were replaced: two subjects in 5 vs. 10, one in 10 vs. 10, and two in 5 vs. 5 contrasts.

## Results

### Experiment 1: Control for swimming activity by manipulating water temperature

In the initial experiment, fish tested at different temperatures showed different overall levels of swimming activity, with the number of cells crossed decreasing with water temperature. Fish in the lower temperature group showed displacements at low speed, whereas at higher temperature fish moved faster (mean ± SEM, 21°C: 88.86 ± 12.21; 25°C: 145.79 ± 18.06; 29°C: 169.36 ± 25.94). Temperature had a significant effect upon fish activity (ANOVA: *F*_2,39_ = 4.475, *P* = 0.018), and the Tukey HSD test confirmed a significantly reduced locomotor activity of fish in the lower temperature group (21°C) relative to that of fish in the higher temperature group (29°C; *P* = 0.016). Activity of fish tested at 25°C was intermediate and not significantly different from that of fish in either of the other groups (*P* > 0.05).

When test fish were placed in a novel test aquarium in the absence of stimulus shoals they generally swam actively mainly along the rear wall of the aquarium. All fish were observed to enter both ends of the test aquarium and during this period they showed a significantly higher swimming activity (number of lines crossed) compared to that shown in the presence of the stimulus shoals (mean ± SEM: 53.07 ± 3.31 and 37.04 ± 2.23, respectively; paired *t*-test: *t*_69_ = 4.528, *P* < 0.001). The reduced shuttling activity during the presence of stimulus shoals is due to experimental fish staying longer in the preference zones close to the stimulus fish. This pattern was similar under the two experimental treatment conditions (10 vs. 5 and 3 vs. 2: overall mean ± SEM: 51.43 ± 4.97 before test and 38.71 ± 3.33 during test; *t*_27_ = 2.216, *P* = 0.035) as well as in the three control situations (10 vs. 10, 5 vs. 5, and 3 vs. 3: overall mean ± SEM: 54.17 ± 4.45 before test and 35.93 ± 3.00 during test; *t*_41_ = 4.038, *P* < 0.001).

**Figure 2:**
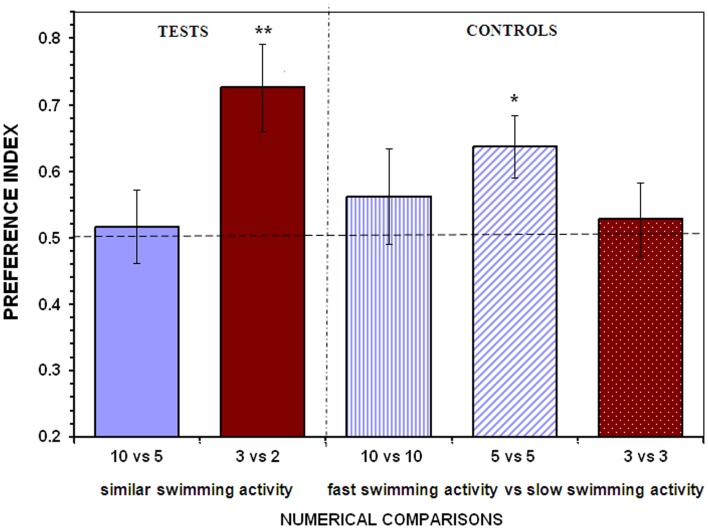
**Control for the overall activity of the contrasting stimulus shoals**. To minimize differences in the overall levels of swimming activity between the shoals, the water temperature was varied. The large shoal was presented in water at 21°C in the stimulus compartment, whereas the smaller shoal at 29°C. The water temperature in the test aquarium, where the test fish were introduced, was at 25°C. In the control treatments one of the equally sized shoals was presented at 29°C and the other at 21°C. Proportion of time (*preference index*) spent by test fish in the 10 cm preference zone close to stimulus fish (mean ± SEM) is shown. Values above 0.5 indicate a preference for the more numerous shoal of stimulus fish or a preference for the more active, faster moving shoal when the stimulus shoals are of the same numerical size. Significant departure from the null hypothesis of no preference is indicated by asterisks: **P < 0.01, *P < 0.05.

When presented with the 10 vs. 5 fish contrast test fish failed to show preference for either shoal (*t*_13_ = 0.297, *P* = 0.771; Figure 2). In this test situation the larger shoal contained slow moving (21°C temperature) and the smaller shoal contained fast moving (29°C temperature) stimulus fish, which made the overall swimming activity of these two contrasted stimulus shoals statistically indistinguishable (mean ± SEM: large shoal 197.50 ± 23.57, small shoal 232.36 ± 22.40; unpaired *t*-test, *t*_12_ = 1.072, *P* = 0.305). Thus, it appears, that when overall swimming activity is similar despite the numerical difference between the contrasted shoals, angelfish were unable to distinguish the two shoals, a result that suggests that indeed angelfish perceives and responds to swimming activity when making a choice between shoals. Interestingly, however, when small shoals (3 vs. 2 fish) were contrasted, experimental angelfish reliably chose the larger shoal (*t*_13_ = 3.420, *P* = 0.005; Figure 2) despite that both shoals had statistically indistinguishable levels of overall activity (mean ± SEM: large shoal 139.71 ± 13.71, small shoal 151.86 ± 19.52; unpaired *t*-test, *t*_12_ = 0.509, *P* = 0.620). Thus, we conclude that activity of the contrasted shoals did not play a significant role when experimental angelfish fish had to discriminate between small quantities. This result was confirmed in the control condition in which small shoals of equal numerical size (3 vs. 3 fish) but with expected different overall levels of swimming activity were contrasted. In this test situation, experimental angelfish did not discriminate the shoals and performed at a level not significantly different from chance (*t*_13_ = 0.497, *P* = 0.627; Figure 2).

In the other control treatments using large shoals, however, we obtained different results. We found experimental angelfish not to show a significant discrimination between shoals of differing activity levels in the 10 vs. 10 fish comparison (*t*_13_ = 0.846, *P* = 0.403; Figure 2) although they still appeared to prefer the faster swimming shoal. However, experimental angelfish did exhibit a significant preference in the 5 vs. 5 fish comparison, spending significantly more time close to the shoal that was kept at the high temperature (29°C, and thus was expected to show increased activity) compared to the other shoal that was kept at the low temperature (21°C, expected low activity; *t*_13_ = 2.890, *P* = 0.013; Figure 2).

### Experiment 2: Control for swimming activity by restricting the space available to fish

As in the former experiment, during the acclimation period with no stimulus shoals, all test fish entered both ends of the tanks and exhibited a significantly higher swimming activity as compared to that shown in the presence of the stimulus shoals (mean ± SEM: 61.11 ± 4.13 and 42.34 ± 3.00, respectively; paired *t*-test: *t*_69_ = 4.35, *P* < 0.001). This pattern was also found in the two treatments in which the stimulus shoals were of different numerical size (mean ± SEM: 57.96 ± 4.61 and 29.32 ± 3.59, respectively; paired *t*-test: *t*_27_ = 7.801, *P* < 0.001), suggesting that during the test period experimental fish stayed close to the stimulus shoals, thus reducing shuttling activity. However, such overall reduction of activity during tests was not significant relative to the acclimation period when the shoals had equal numerical size (10 vs. 10, 5 vs. 5, and 3 vs. 3: overall mean ± SEM: 63.21 ± 6.19 before test and 51.01 ± 3.88 during test; paired *t*-test: *t*_41_ = 1.844, *P* = 0.072). This finding may possibly be due to greater difficulty in decision making by experimental angelfish during the control treatments, resulting in experimental fish moving more frequently from one stimulus shoal to the other.

**Figure 3:**
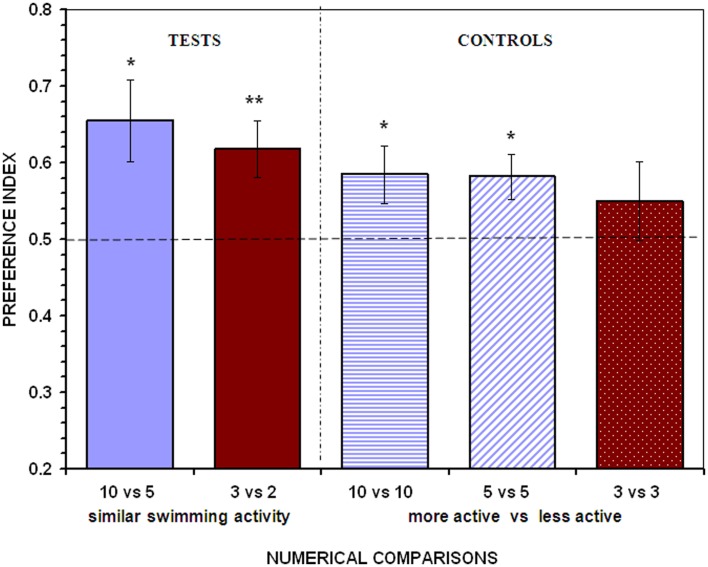
**Control for the overall activity of the contrasting stimulus shoals**. To equate swimming activity between the members of the shoals, the stimulus compartments were divided into 10 small sectors using transparent partitions. Each fish of the shoals was individually confined into each of the adjacent sectors, where activity was restricted. In the case of shoals of five, three, and two fish, these were restrained into each of the central sectors. In the control treatments one of the shoals remained in the small sectors (i.e., stationary) while the other was allowed to swim (i.e., active). Proportion of time (*preference index*) spent by test fish in the 10 cm preference zone close to stimulus fish (mean ± SEM) is shown. Values above 0.5 indicate a preference for the more numerous shoal of stimulus fish or a preference for the active shoal when the stimulus shoals are of the same numerical size. Significant departure from the null hypothesis of no preference is indicated by asterisks: **P < 0.01, *P < 0.05.

When given a choice between two large stimulus shoals (10 vs. 5 fish) in which the movement of the fish within each stimulus was restricted, a significant preference for shoaling with the larger shoal was found (*t*_13_ = 2.892, *P* = 0.013; Figure 3). Likewise, when the two contrasting shoals were numerically small (3 vs. 2 fish) a significant preference for the larger shoal was again detected (*t*_13_ = 3.166, *P* = 0.007; Figure 3). These results suggest that the swimming activity of the shoals is not a fundamental cue when angelfish make shoaling decisions, at least within the range of the numerical size of the shoals and under the experimental conditions employed in this study.

The control experiments, however, suggested that the activity of the shoals did affect shoal preferences in the large number range. When test angelfish were presented with two shoals of identical numerical size in which one of the shoals had quasi-stationary members whereas the other had fish swimming freely, a significant preference for the more active shoal was found in both the 10 vs. 10 fish, and the 5 vs. 5 fish comparisons (*t*_13_ = 2.237, *P* = 0.043; *t*_13_ = 2.788, *P* = 0.015, respectively; Figure 3). In contrast, when the choice was between two small shoals of identical numerical size (3 vs. 3 fish), angelfish performed at a level not significantly different from chance, showing no preference for any of the stimulus shoals (*t*_13_ = 0.967, *P* = 0.351; Figure 3). These control treatments demonstrate that angelfish is sensitive to the activity of the stimulus shoals and that this variable can be an important cue that guides the choice of angelfish when large numbers are involved.

## Discussion

At the origin of the present research is the broader issue of whether fish discriminate between shoals of conspecifics of different size on the basis solely of number or they respond to continuous variables that co-vary with number. In preceding studies we found that angelfish preferred the larger stimulus shoal to the smaller one both when large shoals (10 vs. 5 fish) and when small shoals (3 vs. 2) were contrasted (Gómez-Laplaza and Gerlai, 2011b, 2013 in press). But in these experiments we did not control for the potential influence of swimming activity of the stimulus shoals. The present experiments were designed to examine the potential role of swimming activity, a prominent non-numerical cue. Experiment 1 showed that when large shoals were contrasted (10 vs. 5) and the difference between overall activity level in the numerically different shoals was minimized, angelfish showed no preference for the larger shoal. In contrast, when comparisons involved small shoals (3 vs. 2) fish did prefer the larger shoal even when potential differences in activity levels were minimized between the contrasted stimulus shoals. This latter result was confirmed in Experiment 2 using a different method of controlling the influence of activity (by restricting the movement of fish in the stimulus shoals). Again fish exhibited a preference for the larger shoal (three fish) over the smaller one (two fish). In summary, we found that overall activity of stimulus shoals had no significant influence on the decision making of angelfish when selecting between two shoals within a small number range. Further support for these results comes from the outcome of the control treatments. Neither in Experiment 1 nor in Experiment 2 did we find a significant preference for the stimulus shoal (3 vs. 3 fish) that was expected to be more active (because of warmer water, or more freedom to swim around). Thus, we conclude that when it comes to comparing shoals of small size, overall activity level of the contrasted shoals is not a necessary characteristic upon which angelfish base their discrimination. It is notable that although the preference did not reach significance (control treatments), it is possible that with a larger sample size, and a greater statistical power, the tendency would have been found significant, a question that needs further clarification in the future.

One could argue that even for small shoal comparisons angelfish might have used other continuous cues. However, our previous findings already ruled out the potential role of density, inter-fish distance, and space occupied by the stimulus shoals (Gómez-Laplaza and Gerlai, 2011b, 2013 in press). Others studying another fish species, the mosquitofish, have also showed that overall swimming activity does not affect discrimination between small shoals (Agrillo et al., 2008b). Furthermore, using the method of sequential presentation of the fish, Dadda et al. (2009) reported that density and the proportion of space occupied by the shoals did not affect preference, and using a training procedure with geometric figures Agrillo et al. (2009) found that density of the elements, total luminance, or the sum of perimeters of the stimuli did not affect performance in mosquitofish. Thus, it appears that when discriminating between small shoals fish do not use some prominent continuous variables. Nevertheless, other non-numerical variables such as overall space occupied and cumulative surface of the sets of geometric figures (Agrillo et al., 2009), as well as the surface area of the stimulus fish (Agrillo et al., 2008b) were found to influence discrimination of small quantities. In different animal species, surface area (or cumulative amount) has also been shown to provide a basis for discrimination (e.g., Stevens et al., 2007; Beran et al., 2008; Tomonaga, 2008; Pisa and Agrillo, 2009), and studies with human infants indicate that they may rely on surface area or contour length when discriminating between small quantities (Clearfield and Mix, 1999; Feigenson et al., 2002; Xu, 2003; Cordes and Brannon, 2008). Thus, surface area (and/or contour length) seems to be a salient stimulus property affecting discrimination in several species including fish. We have not tested the potential influence of the surface area of the stimulus shoals in angelfish preference, but at present, after controlling for a number of non-numerical variables, one at a time, (i.e., inter-fish distance, linear extent, density, and now swimming activity of the stimulus shoals) our results indicate that at least these variables have little effect on angelfish’s discrimination of small shoals. Nevertheless, further research is needed to assess the importance of other stimulus properties, particularly surface area and boundary length of the stimuli. Furthermore, we may also need to control for all confounding variables simultaneously to conclusively ascertain the capacity of angelfish to utilize number representation when small shoals are encountered.

Although we found angelfish not necessarily to show discrimination between small shoals on the basis of overall activity levels of the shoals, when large shoals were contrasted the results yielded a different picture. As noted earlier, in Experiment 1 with the potential differences in level of activity minimized between the shoals, subjects were not able to distinguish between shoals of 10 vs. 5 conspecifics. Preference for the more active shoal apparently increased but did not reach significance when the number of fish in the shoals was equated (10 vs. 10). Swimming activity of the stimulus shoals was also found to influence discrimination in the other control treatment (5 vs. 5). Here experimental angelfish did exhibit a significant preference for the more active of the two equally sized shoals. We can conclude from these results that the overall activity difference can be a sufficient condition for discrimination in angelfish, and it seems to be a necessary condition as indicated by the results when the overall difference in swimming activity between the numerically different shoals was minimized (10 vs. 5). These results are fairly consistent with the discrimination of large shoals found in mosquitofish. In this latter species, subjects responded at chance level when presented with shoals of four vs. eight individuals as long as swimming activity was equated between shoals through manipulation of water temperature (Agrillo et al., 2008b). Salamanders, another simple vertebrate, have also been reported to discriminate between large quantities on the basis of movement (Krusche et al., 2010). Furthermore, other continuous variables such as density (Frommen et al., 2009) and surface area (Agrillo et al., 2008b) have also been shown to play a role in discrimination of large shoals in fish. Cumulative surface area also affected discrimination of large quantities of geometric figures in mosquitofish (Agrillo et al., 2010). Although in a previous study neither overall space occupied by the shoals nor inter-fish distance were found to be necessary conditions for the angelfish’s choice between large shoals (10 vs. 5), density was shown to play a role in the choice (Gómez-Laplaza and Gerlai, 2013 in press). It is possible that the denser 10 fish shoal relative to the 5 fish shoal had a potentially greater overlap among individuals, reducing the visibility of the shoal, and the test fish thus could not have perceived it as large enough to be selected. This seems unlikely since fish in the 10 fish shoal were moving slowly and the formation of denser shoals tends to be greater at higher temperatures (see below). Therefore, the role played by density and swimming activity (current experiment) indicates that angelfish are not necessarily able to discriminate large quantities on purely numerical basis. Interestingly, in some fish species it has been reported that, after controlling for non-numerical variables, individuals are able to discriminate between large quantities, apparently with number controlling the selection (Agrillo et al., 2010; Bisazza et al., 2010), but the use of some non-numerical variables could not completely be excluded. Results with human infants and non-human primates suggest that these subjects rely mainly on number when discriminating large sets (Lipton and Spelke, 2003; Brannon et al., 2004; Xu et al., 2005), even though the sets were composed of moving items (Beran, 2008; Beran et al., 2011).

We have not controlled for all continuous variables simultaneously, and also have not systematically examined the potential effect of surface area and contour length. Also notably, in one of our control conditions, the one in which we presented two 10-member fish shoals kept at low vs. high water temperatures (inactive vs. active), preference for the more active shoal was apparent but did not reach significance. It is not clear why angelfish were unable to make a significant choice under this condition and why they could show a preference for the more active shoal in the 5 vs. 5 condition. As mentioned above, it is possible that larger sample sizes would have allowed us to find this apparent effect significant. Another possible explanation for lack of significance is that at the higher temperature, with fish moving faster within the shoal, individuals can temporarily overlap with each other and may not be always simultaneously visible. The effect of overlap is likely to be greater in larger shoals than in smaller ones thus resulting in different outcomes for the 10 vs. 10 and 5 vs. 5 contrasts. In other words, due to the greater overlap, choice could be affected by the total surface area of the fish which could have been reduced in the more active shoal. A smaller overlap of individuals in the low temperature shoal allowing for all fish to be distinguished from each other appears to be a prerequisite for optimal discrimination, even if discrimination is not based on density perception (see Kramer et al., 2011). The existence of conflicting preferences (e.g., more active shoal with reduced overall surface area at 29°C vs. greater surface area shoal with low swimming activity at 21°C) has been demonstrated to lead to individual variation in discrimination and lack of clear choice (e.g., Wong and Rosenthal, 2005). Until the effect of the surface area with two fully visible shoals is evaluated in angelfish the above arguments remain speculative. Alternatively, it is also possible that fish in the shoals adopted different spatial configuration which could affect discrimination. It has been shown that some fish species increase shoal cohesion and form more compact shoals at higher water temperature (Weetman et al., 1998). Although such shoals may be preferred because they are expected to provide better protection from potential danger, aggregating closely may also indicate greater potential predation risk (i.e., it is an antipredator behavior, Gotceitas et al., 1995; Speedie and Gerlai, 2008) and these conflicting cues could restrain test fish from clearly selecting the more active shoal. Thus, the potential benefit of an active shoal may be outweighed by the potential cost of increased risk exposure and this could affect selection of shoal. Position in the water column, postural changes, or other more subtle behavioral differences between shoals could likewise affect the spatial configuration of the shoals and provide cues that influence the decision making of fish, as it has also been suggested for other animal species (e.g., Kilian et al., 2003; Beran, 2006; Krusche et al., 2010). Specific experimental studies are needed before a precise explanation of the behavior exhibited by test fish in this condition can be offered.

In contrast to the results of Experiment 1, Experiment 2 suggests that a clear choice between numerically different shoals is exhibited even when differences in activity of these shoals is minimized. Angelfish were capable of assessing differences in shoal size, preferring the larger shoal (10 fish) over the smaller one (5 fish) even though the activity level of both shoals was similar. Although linear extent of the contrasted shoals was different, this continuous variable has previously been shown not to have much influence on shoal choice (Gómez-Laplaza and Gerlai, 2013 in press). Notably, however, surface area also differed between the above shoals and effect of this variable has not yet been tested. It is also notable that when confronted with shoals of equal size (10 vs. 10, and 5 vs. 5), with one of the two confined to a small space (leading to reduced activity) and the other not (high level of activity), angelfish did spend significantly more time near the active shoal, revealing that angelfish are able to use activity level as a cue in their choice. These results suggest that the activity level, as controlled in this experiment, may contribute to discrimination and that it can be a sufficient cue with large shoal size (see discrimination between more and less active shoals in case of 10 vs. 10 and 5 vs. 5 contrasts). However, the possibility exists that restraining the stimulus fish provides some yet uninvestigated cue that experimental subjects may perceive. For example, fish in the restrained condition could be more stressed than freely swimming fish and this could favor the approach to the more active shoal. Although we did not observe any particular postural or body coloration change or other signs of stress in the restrained fish, some subtle changes could have been perceived by the subjects and could potentially affect the choice made by the experimental subjects. In several animal species, it has been shown that restrained conspecifics may elicit withdrawal rather than approach. Although approach of restrained conspecifics, in order to explore their state, is also observed, this is often a short-term behavior as compared to the longer lasting approach to and preference for free moving conspecifics (e.g., see Watanabe, 2012). These complex effects will be evaluated in the future and may better illuminate our results obtained in the control treatments with large shoal sizes in Experiment 2. Notably, however, one may expect such features of the restrained shoals to also occur in the 3 vs. 3 contrasts, but in this case no significant preference was exhibited by angelfish. Clearly, further research is needed to disentangle these possible explanations.

It is also possible that fish used swimming activity in combination with inter-fish distance, since shoals could also differ in this latter variable. More active shoals may be particularly important in natural situations. Shoals containing fast swimming fish may be preferred because activity levels can indicate increased chances of finding food or anticipation of food (Reebs and Gallant, 1997), and therefore may convey fitness benefits. The ability to quantify moving stimuli as opposed to stationary stimuli has also been shown in primates (e.g., Beran, 2008).

Considering findings published in the literature as well as these above results, it is likely that angelfish as well as other species can base their discrimination upon several attributes of the contrasted stimulus sets. These can include actual number, continuous variables and/or combination of certain continuous features, and numerical attributes. It is also likely that individuals may preferentially rely on one or another such factor depending upon task and context. In summary, further investigation of the relations among these variables is needed. Additionally, our results also underscore what Agrillo and Miletto Petrazzini (2012) stated. These authors argued that it is important to assess different methods and to obtain replication of results. Application of these different approaches will help us better understand the perceptual and cognitive mechanisms that underlie context-dependent differences observed in quantity discrimination and numerical competence across a variety of species.

## Conflict of Interest Statement

The authors declare that the research was conducted in the absence of any commercial or financial relationships that could be construed as a potential conflict of interest.
